# Long-Term Outcome of Fat Grafting to Treat Facial Systemic Sclerosis: A Prospective Cohort Study

**DOI:** 10.1097/PRS.0000000000012111

**Published:** 2025-03-25

**Authors:** Aurora Almadori, Michelle Griffin, Jeon Hyun, Esther Hansen, Christopher P. Denton, Peter E. M. Butler

**Affiliations:** London, United Kingdom; From the ^1^Centre for Nanotechnology and Regenerative Medicine, Division of Surgery and Interventional Science, University College London; 2 Department of Plastic Surgery; 3 Charles Wolfson Centre for Reconstructive Surgery; 4 Clinical Psychology, Department of Plastic Surgery; 5 Center for Rheumatology, Royal Free London NHS Foundation Trust Hospital.

## Abstract

**Background:**

Systemic sclerosis (SSc) is characterized by subcutaneous tissue loss and dermal fibrosis, with limited facial movement and mouth opening. Fat grafting is a minimally invasive technique used to restore facial volume and improve skin fibrosis.

**Methods:**

A cohort of 93 patients were assessed using 3-dimensional imaging before and after fat grafting. Secondary outcomes included physician-based assessment; mouth function (using the Mouth Handicap in Systemic Sclerosis Scale), psychological status, quality of life (using the Derriford Appearance Scale, Hospital Anxiety and Depression Scale, visual analog scale, and Brief Fear of Negative Evaluation Scale); and patient satisfaction.

**Results:**

After an average of 2.96 ± 2.2 sessions of fat grafting, with an injection volume of 11.9 ± 6 cc in each session, the overall retention rate was 53.1% ± 0.17% at an average follow-up of 3.11 ± 1.73 years. Patients undergoing 5 or more interventions presented a higher retention rate (73.1% ± 0.08%) than those receiving 1 or 2 treatments (45.2% ± 0.09% and 50.5% ± 0.15%, respectively) (*P* < 0.05). Significant improvements were found in mouth function (*P* < 0.0001) and quality of life (*P* < 0.0001).

**Conclusions:**

Facial fat grafting is an effective technique for restoring facial volume and improving oral function and quality of life in patients with scleroderma. This study presents the largest number of patients published to date with the longest follow-up period.

**CLINICAL QUESTION/LEVEL OF EVIDENCE:**

Therapeutic, IV.

Systemic sclerosis (SSc) is a chronic autoimmune disease characterized by abnormal deposition of extracellular matrix with progressive fibrosis.^1,2^ It is classified into 2 subsets: limited cutaneous SSc (lcSSc), if the skin fibrosis is confined to the face and extremities, and diffuse cutaneous SSc (dcSSc), if the skin fibrosis extends on the trunk and proximal parts of the limbs.^1,2^

Skin fibrosis and subcutaneous tissue loss are hallmark manifestations that are particularly noticeable in the face. Thickened and hardened skin is responsible for a taut and mask-like appearance with diminished facial expression. Typical orofacial features include subcutaneous tissue loss, fibrotic skin firmly attached to the underlying planes, nasal alar resorption resulting in a pointed nose, perioral wrinkles, and mouth changes.^2^ The lips are usually thinned (microcheilia), and the skin around the mouth is stretched with deep perioral radial wrinkles, loss of the vermilion border of the lips, and narrowing of the oral line with reduced motility and opening (microstomia).^2^ When salivary gland function is impaired, dryness of the oral mucosa can be a feature (xerostomia).^2,3^

Life-threatening aspects of the disease (heart, lung, and renal involvement) have been a therapeutic priority, but nonlethal manifestations, such as facial and hand impairments, are important,^4^ because patients with scleroderma experience impaired quality of life, poor mental health, self-image issues, depression, anxiety, and lower perception of general health compared with healthy controls.^2,4–7^

Fat grafting is minimally invasive surgery largely used in plastic surgery not only to fill contour deformities but also to improve skin fibrosis and scarring.^2,8–11^ Its use in scleroderma has been successful in correcting volumetric deformities in localized scleroderma^12–15^ and improving mouth or hand function in SSc.^16–21^ However, studies published thus far present a short follow-up period; therefore, data on the durability of the effect in facial scleroderma are lacking. Evidence of the use of lipotransfer for other applications has shown that it is associated with unpredictable long-term results due to volume resorption, which is the main limitation of this technique.^22,23^

This prospective cohort study aimed to assess the long-term volumetric outcomes after fat grafting in patients with SSc.

## PATIENTS AND METHODS

### Trial Design

This study included 93 patients who met our inclusion criteria: previous diagnosis of SSc, irrespective of the disease subset (dcSSc and lcSSc); age from 18 to 75 years; stabilized SSc disease (at least 2 years); stable lung and cardiac functionality; noticeable orofacial modification; and verified orofacial dysfunction, assessed with the Mouth Handicap in Systemic Sclerosis scale. Exclusion criteria were pregnancy, widespread infection or cancer, or inability to give written informed consent in English.

Patients were selected from the Rheumatology and Plastic Surgery outpatient clinics. They were offered repeated procedures based on their clinical signs and symptoms with 12-month intervals between the procedures.

### Ethics Approval

This prospective study was conducted after approval from institutional and regional ethics committees (REC Hampshire B reference 16/SC/0669; R&D reference 10006).

### Surgical Technique

Fat was harvested using 15 × 3-mm disposable cannulas with 2 holes of 1 mm diameter. Donor sites were abdomen or medial thighs, where a sufficient amount of fat is usually present irrespective of the patient’s disease severity or body mass index. Lipoaspirate was centrifuged at 3000 rpm for 3 minutes.^8^ We used only the distal three-fourths of the lipoaspirate, because centrifugation differentially concentrates adipose-derived stem cells (ASCs) in this fraction of the lipoaspirate.^24^ The injecting cannula had a 3-mm diameter and was inserted through a skin incision or intraorally with access through the mucosa. Small aliquots of fat were deposited linearly on the recipient sites, including the lips, nose (dorsum and alae), cheeks, and chin.

### Outcome Measures

Patients were prospectively assessed before and after treatment. Preoperative assessment was performed during outpatient appointments or on the day of surgery before the operation. Postoperative assessments were performed at 12 months and at successive outpatient appointments at 12-month intervals. The assessment included facial volume with 3-dimensional (3D) imaging, mouth function, psychological status and quality of life, physician-based evaluation, and patient-based satisfaction.

#### Facial Volumes and Fat Graft Survival Rate

Preoperative and postoperative 3D photographs were taken using the static 3dMD system. Patients were scanned while sitting at 90 degrees and instructed to adopt a relaxed facial expression, with their lips resting and their teeth lightly in contact.^25^ Volumetric differences were analyzed using the Vultus software. Pretreatment and posttreatment images were superimposed. To ensure alignment precision, a root mean square error, which indicates the differences between the 2 surfaces’ root mean square, of 0.5 mm maximum was considered acceptable, as per the manufacturer guidelines (www.3dmd.com). After the images were superimposed, the aesthetic units of interest (cheeks, nose, upper lip, lower lip, and chin) were marked using fixed anatomic landmarks, and the volume difference between the preoperative and postoperative images was calculated with the volume measurements function. Following a comparison of the injected volume and the volumetric difference between the 2 surfaces (volume detected), the percentage of volume retained over time was computed.

#### Mouth Function

Mouth disability was assessed with the Mouth Handicap in Systemic Sclerosis scale,^26^ a validated scale with 12 items each scored 0 to 4, with a total score ranging from 0 (minimal handicap) to 48 (maximal handicap). The 12 items are divided in 3 domains: mouth function, mouth dryness, and aesthetic concerns.^26^

#### Psychological Status and Quality of Life

The psychological aspect and quality of life were assessed using multiple validated measures. The Derriford Appearance Scale measures the degree of psychological distress associated with physical appearance.^27^ The Hospital Anxiety and Depression Scale is a validated self-report questionnaire that identifies and quantifies anxiety disorders and depression.^28^ The noticeability of the disfigurement measure consists of 3 visual analog scales (VAS) from 0 to10 in which the individual ranks the self-perceived noticeability of the disfigurement.^29,30^ The Brief Fear of Negative Evaluation Scale measures social anxiety disorder,^31^ and is composed of 12 items related to worry or fearful cognition.^32^

#### Physician-Based Satisfaction

Preoperative and postoperative photographs of each patient were assessed and graded as improved or not improved. Improvements were further graded as minor or substantial. Improvement was defined as the overall facial volume restoration, orofacial disease severity, and mouth appearance.

#### Patient-Based Satisfaction

At the final follow-up assessment, patients were asked to answer general questions regarding their satisfaction with the procedure.

### Patient and Public Involvement

Patients with SSc actively participated in the selection of patient-reported outcome measures in this study to determine which features were more pertinent to them.

### Statistical Analysis

Volumetric changes in fat grafts over time are presented as percentages. Intercomparisons between pretreatment and posttreatment values were analyzed statistically using a paired *t* test with a nonparametric Wilcoxon matched pairs signed rank test (Prism6 Software). Unrelated groups (ie, lcSSc versus dcSSc) and groups presenting different variables (ie, different numbers of treatments) were analyzed using nonpaired *t* tests (Prism6 Software). The tests were 2-tailed, with a confidence interval of 95%. Means and standard deviations were calculated. Statistical significance was set at *P* < 0.05.

## RESULTS

### Demographics

Of the 93 included patients, 98% were female. Average age at the time of surgery was 52 ± 1.46 years. Average disease duration was 12.27 ± 7.95 years. There was a nearly equal distribution of disease subsets, with 46% (*n* = 43) diagnosed with dcSSc and 54% (*n* = 50) with lcSSc. In addition, 63% of the patients (*n* = 59) had concurrent immunosuppression at the time of surgery and 38% (*n* = 34) did not. Overall, 89% of the patients (*n* = 83) presented with specific autoantibodies, and 30% (*n* = 28) were associated with additional overlap syndromes (Table 1).

**Table 1.** Demographic Data

Characteristics	Values
Total no. of patients	93
Sex, no. F/M	91/2
Mean age at time of surgery, yrs	51.70 ± 11.46
Mean duration of disease at time of surgery, yrs	12.27 ± 7.95
Subset, no.	
DcSSc	43
LcSSc	50
Presence of autoantibodies, no.	
ANA-negative	3
ANA-positive, ENA negative	3
Specific autoantibodies	83
Anti-Scl-70	26
Anti-RNAP III	26
ACA	13
Anti-PM-Scl	5
Anti-RNP	3
Anti-PL7	3
Anti-CCP	3
Other	5
Overlapping syndromes, no.	
Antiphospholipid syndrome	1
Systemic lupus erythematosus	3
Sjögren syndrome	7
Rheumatoid arthritis	3
Myositis	7
Inflammatory arthritis	4
Antisynthetase syndrome	2
Vasculitis	1
Concurrent immunosuppression, no.	
No	34
Yes	59
1 immunosuppressant	42
2 immunosuppressants	16
Major drug treatment, no.	
Mycophenolate mofetil	36
Hydroxychloroquine	23
Rituximab	3
Azathioprine	3
Methotrexate	8
Hydroxycarbamide	1

ACA, anti–centromere antibody; anti-CCP, anti–cyclic citrullinated peptide; anti-hnRNP, anti–heterogenous nuclear ribonucleoproteins; anti-PL7, anti–threonyl–tRNA synthetase antibody; anti-PM-Scl, anti–exosome antibody; anti-RNAP III, anti–RNA polymerase III antibody; anti-RNP, anti–ribonucleoprotein antibody; anti-Ro, Anti-La, anti–Sjögren syndrome–related antigen A and B; anti-Scl-70, anti–topoisomerase I antibody; anti-Th/To, antibodies to Th/To ribonucleoprotein; anti-U3RNP, anti–fibrillarin antibody; dcSSc, diffuse cutaneous systemic sclerosis; lcSSc, limited cutaneous systemic sclerosis.

### Operation Details

In total, 275 procedures were performed. Each patient received an average of 2.96 ± 2.2 treatments. For each procedure, 11.9 ± 6 cc of fat was injected into the facial area (Table 2). Different aesthetic units were targeted, as follows: cheeks (3.8 ± 2.4), nose (1.8 ± 0.8), upper lip (2.9 ± 1.4), lower lip (2.6 ± 1.1), and chin (2.0 ± 0.9) (Table 2). Three patients presented moderate abdominal bruising that resolved within 14 to 21 days, and 2 developed postoperative wound infections respondent to oral antibiotic therapy. The average follow-up was 3.11 ± 1.73 years.

**Table 2.** Operation Details and Fat Graft Mean Survival Rate^a^

Aesthetic Unit	Mean Volume Injected, cc	Mean Volume Detected, cc	Survival Rate, %
Face overall	11.9 ± 6.0	6.3 ± 2.5	53.1
Cheeks	3.8 ± 2.4	1.8 ± 1.1	48.5
Nose	1.8 ± 0.8	0.7 ± 0.3	39.4
Upper lip	2.9 ± 1.4	1.4 ± 0.9	50.3
Lower lip	2.6 ± 1.1	1.5 ± 0.7	56.5
Chin	2.0 ± 0.9	1.2 ± 0.7	59.5

a Injected volumes and volumetric analysis performed. For each aesthetic unit, the volume injected and the volume difference detected by comparing the preoperative and postoperative 3-dimensional scans were compared. Volume analysis showed an overall good survival rate of the fat grafted in the face, although the percentage of fat survival varies in different aesthetic units.

### Clinical Outcome

#### Volumetric Outcome and Fat Graft Survival Rate

Volumetric analysis showed that all facial aesthetic units retained a proportion of the injected volume to a lesser or greater extent, with an overall facial survival rate of 53.1% ± 0.17% at a follow-up of 3.11 ± 1.73 years. Chin and lower lip presented the highest percentage of survival (59.5% and 56.5%, respectively). The nose had the lowest survival rate (39.4%), followed by the upper lip (50.3%) and cheeks (48.5%) (Table 2). The statistical significance (*P* values) of the difference in fat survival among the facial aesthetic units is reported in Table 3.

**Table 3.** Statistical Differences (*P* Values^a^) in the Percentage Retention Rate among the Different Facial Aesthetic Units

Aesthetic Unit	Cheeks	Nose	Upper Lip	Lower Lip	Chin
Cheeks	—	0.0011^b^	0.5970	0.1169	0.0184^b^
Nose	0.0011^b^	—	0.0095^b^	0.00001^b^	0.000001^b^
Upper lip	0.5970	0.0095^b^	—	0.0531^b^	0.0081^b^
Lower lip	0.1169	0.00001^b^	0.0531^b^	—	0.3785
Chin	0.0184^b^	0.000001^b^	0.0081^b^	0.3785	—

a Unpaired *t* test.

b Statistically significant differences.

Subset analyses were performed according to the patient demographics. (**See Table, Supplemental Digital Content 1**, which shows the effect of demographics on fat graft survival rate. The table illustrates the survival rate [%] taking into consideration the demographic data. Results are represented in the different disease subsets [lcSSc versus dcSSc], in the different concurrent treatment subgroups [immunosuppression versus no immunosuppression], in the different numbers of lipofilling received, in the different age groups, in the presence of autoantibodies, and in the different lengths of disease duration, https://links.lww.com/PRS/H996.) Significantly improved survival rates were found in patients who underwent 5 or more fat grafting procedures (73.1% ± 0.08%) compared with those who underwent 1 or 2 (45.2% ± 0.09% and 50.5% ± 0.15%, respectively; *P* < 0.05). No significant difference in fat survival rates was found between the disease subset (53.9% ± 0.17% versus 52.3% ± 0.16%; *P* = 0.44) and patients on concurrent immunosuppression compared with those not on immunosuppressive therapy (53.3% ± 0.17% versus 52.7% ± 0.16%; *P* = 0.58). The presence of autoantibodies did not confer a significant difference in fat survival rates (*P* = 0.330). Disease duration did not affect survival outcomes (*P* = 0.876).

#### Mouth Function Outcome

A significant improvement in mouth function (*P* < 0.0001) was reported, with a median score of 28 (IQR, 25 to 33) before and 23 (IQR, 20 to 26) after surgery. The mouth opening subset represented 48% of the total mouth score improvement, followed by mouth dryness (33%) and aesthetic concerns (19%) (Table 4).

**Table 4.** Effect of Fat Grafting on Mouth Function Outcome^a^

Mouth Assessment	Median Score before Fat Graft (IQR)	Median Score after Fat Graft (IQR)	Median Score Change (IQR)	*P*
MHISS overall	28 (25–33)	23 (20–26)	5 (2.72–7.5)	0.0001
Mouth opening	13 (10–14)	9 (8–11)	3 (1–4)	0.0001
Mouth dryness	10 (8–13)	9 (6–10)	1 (0–3)	0.0001
Aesthetic concern	7 (5–8)	5.27 (4–6)	1 (0–2)	0.0001

a Data are reported as mouth function overall and subdivided by each domain of the Mouth Handicap in Systemic Sclerosis (MHISS) scale. Paired *t* test (nonparametric Wilcoxon matched pairs signed rank test, Prism6 Software) was performed.

#### Psychological Status and Quality of Life Outcomes

Patients reported a significant improvement in their psychological status following fat grafting (Table 5) in physical appearance (Derriford Appearance Scale) (*P* < 0.0001), self-perceived noticeability of disfigurement (VAS 1) (*P* < 0.0001), perception of how noticeable the disfigurement was to other people (VAS 2) (*P* < 0.0001), worrying about the noticeability of disfigurement (VAS 3) (*P* < 0.0001), anxiety (*P* < 0.0001), depression (*P* < 0.0001) (Hospital Anxiety and Depression Scale), and social anxiety (Brief Fear of Negative Evaluation Scale) (*P* < 0.0001).

**Table 5.** Effect of Fat Grafting on Psychological and Quality of Life Outcomes

Item	Median Score before Fat Graft (IQR)	Median Score after Fat Graft (%)	Median Score Change (%)	*P* ^a^
DAS	47 (38–59)	40 (31–49)	6 (2–14)	0.0001
HADS-A	12 (9–17)	10 (8–12.5)	2 (0–4)	0.0001
HADS-D	11 (7–17)	8 (6–12)	1 (–0.5–5)	0.0001
VAS 1	8 (6–10)	7 (5–8)	1 (0–3)	0.0001
VAS 2	8 (6–10)	6 (5–8)	1 (0–3)	0.0001
VAS 3	9 (7–10)	7 (5.5–8)	1 (0–2.5)	0.0001
BFNES	36 (30–40.5)	32 (28–39)	3 (0–7)	0.0001

BFNES, Brief Fear of Negative Evaluation Scale for social anxiety; DAS, Derriford Appearance Scale for satisfaction with appearance; HADS-A, Hospital Anxiety and Depression Scale–Anxiety; HADS-D, Hospital Anxiety and Depression Scale–Depression; VAS, visual analog scale for noticeability of disfigurement.

a Paired *t* test (nonparametric Wilcoxon matched pairs signed rank test, Prism6 Software) was performed.

#### Physician-Based Assessment

**Fig. 1.**
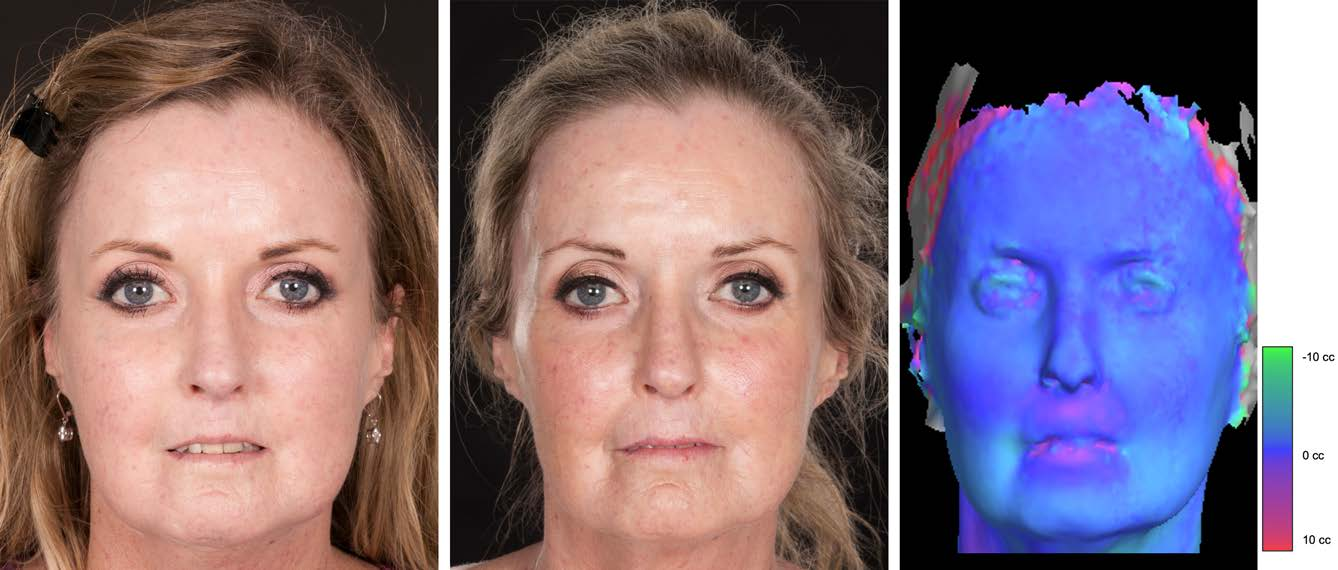
Cosmetic improvement after fat grafting in patient 1. Representative patient with SSc (dcSSc subset). At the time of the first treatment, the patient was 44 years old, and the disease duration was 22 years. The main features were microstomia, microcheilia, overall subcutaneous tissue loss, taut facial skin adherent to the underlying planes, and diminished facial expression (mask-like aspect). The shiny skin appearance is due to the skin being pulled taut over the underlying bone (*left*). The patient underwent 3 fat grafting procedures, and the average amount of subunit injection was (in mL) 2 in the cheeks, 2.17 in the nose, 4.83 in the upper lip, 4 in the lower lip, and 2 in the chin. After treatment, the overall skin became more elastic, resulting in a more relaxed facial expression. The lips showed increased thickness and mouth closure was improved (*center*). The color map generated using the 3dMD system confirmed volumetric enhancement in the perioral area after surgical treatment with fat grafting (*right*).

**Fig. 2.**
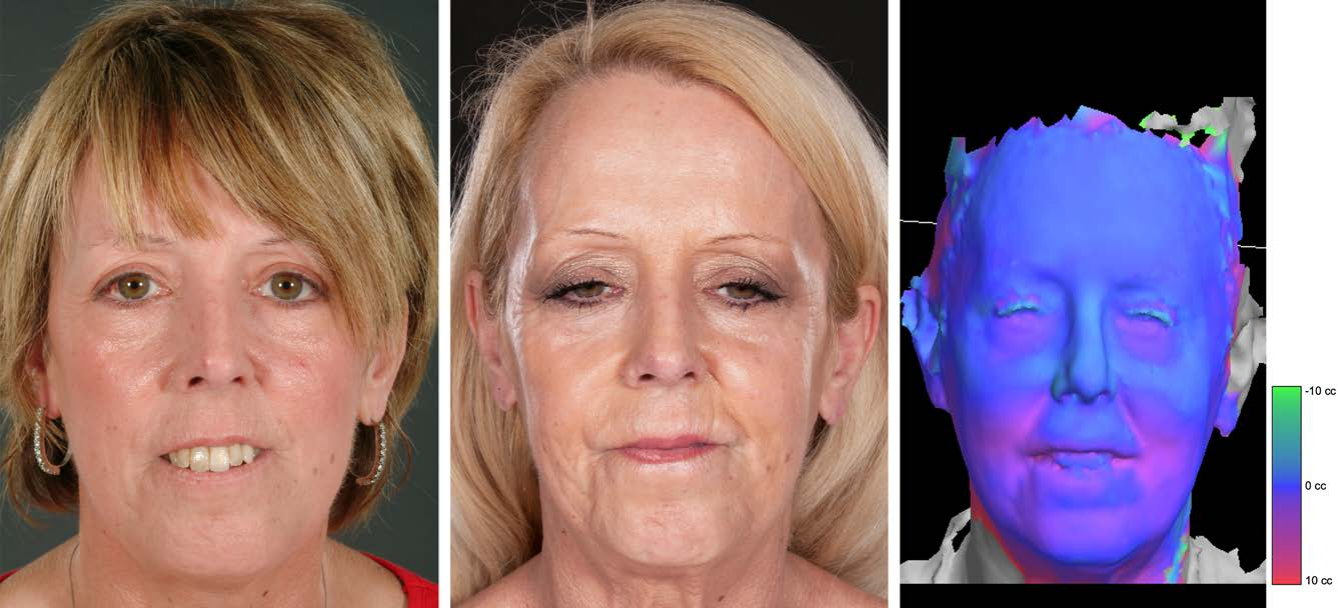
Cosmetic improvement after fat grafting in patient 2. Representative patient with SSc (dcSSc subset). At the time of the first treatment, the patient was 64 years old, and the disease duration was 18 years. The patient presented with microcheilia, microstomia, loss of the vermilion border of the lips, and resorption of subcutaneous tissue in the dorsum and alae of the nose, resulting in a typical scleroderma-pointed nose (*left*). The patient received 9 fat grafting procedures, and the average amount of subunit injection was (in mL) 3.57 in the cheeks, 1.50 in the nose, 3.61 in the upper lip, 3.04 in the lower lip, and 2.19 in the chin. After treatment, the subcutaneous labial thickness increased, allowing normal mouth closure. The pointed nose was corrected with improved overall nasal appearance (*center*). Volumetric analysis confirmed a change in facial volume after surgical treatment with fat grafting, particularly in the inferior third of the face (*right*).

**Fig. 3.**
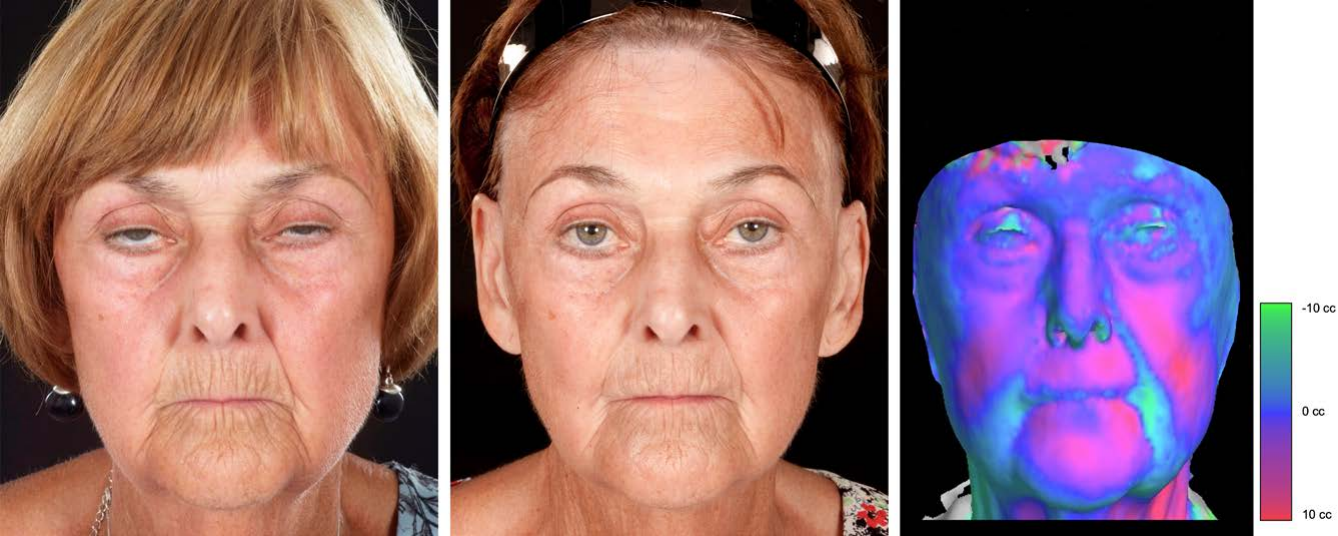
Cosmetic improvement after fat grafting in patient 3. Representative patient with SSc (lcSSc subset). At the time of the first treatment, the patient was 73 years old, and the disease duration was 12 years. In this case, the main characteristics were microcheilia and perioral radial furrows (*left*). After 2 sessions of fat grafting, the marked perioral furrows improved, with increased lip volume and reduced perioral wrinkles (*center*). Average amount of subunit injection was (in mL) 2.83 in the cheeks, 0.8 in the nose, 6.5 in the upper lip, 7 in the lower lip, and 2.5 in the chin. Volumetric analysis showed volumetric augmentation of the cheeks, nose, chin, and lips (*right*).

Out of the 93 patients included, 87% (*n* = 81) were considered improved. Of them, improvement was graded as substantial in 62% (*n* = 50) and minor in 38% (*n* = 31). Figures 1 through 3 illustrate the typical SSc-related facial features before and after fat grafting. These included microstomia and microcheilia (Figs. 1, *left,* and 2, *left*), subcutaneous tissue loss with overall taut facial tissues (Fig. 1, *left*), pointed nose (Fig. 2, *left*), and perioral radial furrows (Fig. 3, *left*), all of which improved after treatment (Figs. 1, *center,* 2, *center*, and 3, *center*).

#### Patient-Based Assessment

At the last follow-up, participants were queried regarding their satisfaction with the procedure: 90% considered their face improved; 83% rated the outcome as good to excellent; 93% would undergo the procedure again; 97% would recommend the treatment to a friend or family member with a similar problem; and 93% reported no complications. The complications reported by patients (7%) were lumps, swelling, and bruising.

## DISCUSSION

In this study, we describe long-term outcomes of fat grafting for correcting facial disfigurement in SSc. The rationale of this treatment is to increase the subcutaneous tissue bulk (volumetric effect) and ameliorate the fibrotic tissues (regenerative effect).^2^ The latter is attributed to the ASC secretome. We previously demonstrated that the surface phenotype and differentiation capacity of ASCs from patients with SSc are identical to those of healthy matched ASCs. The proliferation and migration capacity of ASCs from patients with SSc was reduced, but they were capable of ex vivo culture and expansion.^33^ It has also been demonstrated that ASCs from patients with SSc have functionality similar to that of healthy controls in terms of senescence and mRNA profiles.^21,34^ Previous studies investigating the paracrine effect of ASCs on SSc dermal myofibroblasts found that transforming growth factor β1 and connective tissue growth factor secreted by SSc fibroblasts were significantly reduced when co-cultured with ASCs.^21^ These studies suggest that ASCs play a key antifibrotic role in SSc.

Compared with previous studies on the use of lipofilling in facial SSc,^35–37^ this study presents multiple differences.

First, the treatment was not limited to the perioral area, but was extended to different facial aesthetic units. Patients with scleroderma mainly complain of lip thinning and gradual difficulty in mouth opening; however, patients present with panfacial soft-tissue changes associated with scleroderma, with loss of nasal, cheek, and perioral soft-tissue volume, as well as sclerotic changes to the lips, oral mucosa, and facial skin. Targeting multiple facial aesthetic units achieves more natural results. In fact, we speculated that a paracrine antifibrotic effect might be exerted on the facial skin, allowing increased elasticity and tension release of the overall taut facial tissues, resulting in improved facial movement and a more natural aesthetic outcome. Clear communication with patients is important for managing their expectations and informing them of physiological facial aging. In fact, while scleroderma is characterized by taut and hard skin, after panfacial fat grafting, the replacement of soft tissue and improvement in skin elasticity can make the face more susceptible to normal aging. Therefore, it is important to discuss age-related changes in the facial soft tissues, including descent of the malar fat pad, laxity of the retaining ligaments of the midlateral face, and ptosis within the fascial fatty layer and overlying dermis of the cheek mass (Figs. 1, *center*, and 2, *center*).^38,39^

Second, in this study, smaller amounts of fat were injected compared with previous reports. In the mouth, the average amount of fat graft was on average 5.6 ± 1.3 cc; in previous studies, the range was 16 to 22 cc.^35–37^ Avoiding overcorrection is preferable for obtaining a balanced and long-lasting aesthetic outcome. Considering that the loss of a significant portion of the grafted volume has been the main criticism of this technique,^22^ the injection of small amounts is preferred to maximize the survival rate over time. This is consistent with the work of Eto et al.^40^ and Mashiko and Yoshimura,^41^ who investigated the fate of fat after grafting and proposed the 3-zone principle. We performed smaller-volume injections and, if required, repeated the procedure multiple times, rather than injecting larger volumes at once. Given the severe fibrosis of the skin and underlying tissues in scleroderma, there is often insufficient room in the recipient site to accommodate a larger amount of fat graft. In our series, we demonstrated that the adipose tissue injected in small amounts in multiple procedures survives better over time at an average follow-up of 3.11 ± 1.73 years after the final treatment.

Third, in this study, the average number of treatments received was 2.95 ± 2.11; the other groups reported only 1 treatment. We found that multiple sequential interventions produced a cumulative benefit, and is advisable to achieve a better outcome, with a significantly higher fat survival rate (*P* < 0.05) in patients who received 5 or more fat grafting procedures (73.1% ± 0.08%). Randomized clinical trials are required to provide a clear evidence-based protocol and determine the optimal number of treatments.

We used the standardized fat grafting technique described by Coleman.^8^ Several authors have challenged the value of this technique^42–46^ or mixed it with platelet-rich plasma.^47,48^ However, there is no evidence supporting the superiority of one technique over another in terms of the combined volumetric and antifibrotic effects. Hence, we chose to use the standardized technique to report a series treated with the same technique that did not change over the study period and have a final byproduct that has been fully characterized, allowing future comparison and meta-analysis. Other methods may be explored to optimize the volumetric outcome by enhancing the fat graft survival rate. A recent randomized controlled pilot study on localized scleroderma showed that lipofilling enriched with ASCs presented a higher survival rate than conventional fat grafting and fat grafting enriched with stromal vascular fraction.^49^ However, both ASCs and stromal vascular fraction were obtained through collagenase digestion and are therefore not compliant with the regulations of most countries in the United States and Europe.

### Strengths and Limitations

The main strengths of the study include the objective assessment of the fat survival rate with 3D imaging, a noninvasive and cost-effective tool. Another aspect is the study power. SSc is a rare condition; hence, reports published to date have included small sample sizes. In our series, inclusion of a large cohort of patients was possible because our hospital is the national tertiary referral center for patients with SSc and the largest UK cohort. In addition, this study presents a longer follow-up of an average 3.11 ± 1.73 years versus the 3 to 6 months previously reported.^35–37^ The study presents a strong patient and public involvement component and robust patient-reported outcome measures.

Despite its strengths, this study has limitations. It is a single-arm study without a control group, and therefore a potential placebo effect cannot be excluded. In addition, the study was not blinded, which could have introduced bias in the patient-reported outcomes. In fact, it is well established that when individuals are aware they are being observed, they may change their normal behavior (Hawthorne effect), introducing a risk of bias.^50^

## CONCLUSIONS

This long-term study showed that fat grafting is effective in correcting the facial volumetric and fibrotic changes associated with scleroderma. Despite long-term follow-up, the definitive durability of this effect is unknown, and the optimal number of treatments must be determined, although this may be difficult because scleroderma is a chronic autoimmune disease and the pathological processes driving facial changes are ongoing. Clarification of the mechanism of the antifibrotic action of the technique, through effector cells, multiple cells, or other mechanisms, is of significant interest for the development of an antifibrotic treatment for this and other diseases.

**CODING PERSPECTIVE**

Coding perspective provided by Jeff Kozlow, MD, MS, is intended to provide coding guidance.

15771 Grafting of autologous fat harvested by liposuction technique to trunk, breasts, scalp, arms, and/or legs; 50 cc or less of injectate
+15772 Each additional 50 cc of injectate, or part thereof (list separately in addition to the code for the primary procedure)
15773 Grafting of autologous fat harvested by liposuction technique to face, eyelids, mouth, neck, ears, orbits, genitalia, hands, and/or feet; 25 cc or less of injectate
+15774 Each additional 25 cc of injectate, or part thereof (list separately in addition to the code for the primary procedure)

-  The reporting of autologous fat grafting is straightforward with CPT codes 15771 through 15774.
-  These codes describe the work of harvesting the autologous fat with a liposuction technique, any manual or automated preparation steps, and then the injection of the prepared fat into the recipient area.
  •The direct excision of a piece of adipose tissue that is placed in a surgical defect would be reported with CPT code 15769.
-  Code selection is based on the location of the injection and the volume of fat injected.
  •The volume thresholds are different between the identified areas, with 50 cc used for the larger and less sensitive areas, compared to 25 cc for the smaller and more sensitive areas.
  •The volume of injectate is representative of the volume used to address the recipient area. Any additional volume of fat that is harvested and/or prepared, but not injected, is not reported.
-  Codes 15771 and 15772 are used to report the injection of the autologous fat into the trunk, breasts, scalp, arms, or legs.
  •Code 15771 is used to report up to the initial 50 cc of autologous fat injected, and add-on code 15772 is used to report additional volume in units of up to 50 cc per unit.
  •Medicare has a Medically Unlikely Edit of 9 units for code 15772, meaning that it will not allow reporting of greater than 9 units using code 15772 (although this can be appealed with appropriate documentation). The reporting of codes 15771 and 15772 × 9 units would represent 500 cc of injected fat.
-  Codes 15773 and 15774 are used to report the injection of the autologous fat into the typically more sensitive areas of the face, eyelids, mouth, neck, ears, orbits, genitalia, hands, and feet.
  •Code 15773 is used to report up to the initial 25 cc of autologous fat injected, and add-on code 15774 is used to report additional volume in units of up to 25 cc per unit.
  •Medicare has a Medically Unlikely Edit of 3 units for code 15774, meaning that it will not allow reporting of greater than 3 units using code 15774 (although this can be appealed with appropriate documentation). The reporting of codes 15773 and 15774 × 3 units would represent 100 cc of injected fat.
-  As with the repair codes or the split-thickness skin grafting codes, the volume for reporting codes 15771 through 15774 is additive for all the represented areas. Examples are as follows:
  •If a patient undergoes 10 cc of fat grafting to the perioral area, 3 cc to the right eyelid, and 3 cc to the left eyelid, the appropriate code is 15773 × 1 unit, since the total injectate within the collective anatomic grouping is 16 cc.
  •If a patient undergoes 40 cc of fat grafting to the breast and 15 cc to the perioral area, the appropriate codes are 15771 × 1 unit and 15773 × 1 unit, since the breast and perioral area are listed in separate codes.

**Disclosure:** Jeffrey Kozlow, MD, MS, has no financial disclosures to report. He serves as the American Society of Plastic Surgeons co-advisor to the American Medical Association’s CPT Editorial Panel and Relative Value Scale Update Committee.

## DISCLOSURE

The authors have no financial relationships or conflicts of interest to disclose. No funding was received for this study.

## PATIENT CONSENT

Patients provided written informed consent for the use of their images.
